# Exposure to PM_2.5_ Metal Constituents and Liver Cancer Risk in REVEAL-HBV

**DOI:** 10.2188/jea.JE20220262

**Published:** 2024-02-05

**Authors:** Tzu-Yi Lu, Chih-Da Wu, Yen-Tsung Huang, Yu-Cheng Chen, Chien-Jen Chen, Hwai-I Yang, Wen-Chi Pan

**Affiliations:** 1 Institute of Environmental and Occupational Health Sciences, National Yang Ming Chiao Tung University, Taipei, Taiwan; 2 Department of Geomatics, National Cheng Kung University, Chiayi, Taiwan; 3 Department of Environmental Health, Harvard School of Public Health, Boston, MA, USA; 4 Institue of Statistical Science, Academia Sinica, Taipei, Taiwan; 5 National Institution of Environmental Health Sciences, National Health Research Institute, Mioli, Taiwan; 6 Genomics Research Center, Academia Sinica, Taipei, Taiwan; 7 Graduate Institute of Epidemiology and Preventive Medicine, National Taiwan University, Taipei, Taiwan; 8 Institute of Clinical Medicine, National Yang Ming Chiao Tung University, Taipei, Taiwan

**Keywords:** fine particulate matter, metal constituents, liver cancer, Taiwan

## Abstract

**Background:**

Ambient particulate matter is classified as a human Class 1 carcinogen, and recent studies found a positive relationship between fine particulate matter (PM_2.5_) and liver cancer. Nevertheless, little is known about which specific metal constituent contributes to the development of liver cancer.

**Objective:**

To evaluate the association of long-term exposure to metal constituents in PM_2.5_ with the risk of liver cancer using a Taiwanese cohort study.

**Methods:**

A total of 13,511 Taiwanese participants were recruited from the REVEAL-HBV in 1991–1992. Participants’ long-term exposure to eight metal constituents (Ba, Cu, Mn, Sb, Zn, Pb, Ni, and Cd) in PM_2.5_ was based on ambient measurement in 2002–2006 followed by a land-use regression model for spatial interpolation. We ascertained newly developed liver cancer (ie, hepatocellular carcinoma [HCC]) through data linkage with the Taiwan Cancer Registry and national health death certification in 1991–2014. A Cox proportional hazards model was utilized to assess the association between exposure to PM_2.5_ metal component and HCC.

**Results:**

We identified 322 newly developed HCC with a median follow-up of 23.1 years. Long-term exposure to PM_2.5_ Cu was positively associated with a risk of liver cancer. The adjusted hazard ratio (HR) was 1.13 (95% confidence interval [CI], 1.02–1.25; *P* = 0.023) with one unit increment on Cu normalized by PM_2.5_ mass concentration in the logarithmic scale. The PM_2.5_ Cu-HCC association remained statistically significant with adjustment for co-exposures to other metal constituents in PM_2.5_.

**Conclusion:**

Our findings suggest PM_2.5_ containing Cu may attribute to the association of PM_2.5_ exposure with liver cancer.

## INTRODUCTION

Outdoor particulate matter was classified as a carcinogen to humans by the International Agency for Research on Cancer (IARC) in 2013.^1^ Based on United States and European populations, long-term exposure to fine particulate matter (PM_2.5_) is consistently associated with an increased risk of lung cancer.^2^^–^^5^ Observational studies showed associations of ambient air pollutants with cervical cancer,^6^ brain cancer,^6^^,^^7^ as well as bladder cancer,^8^^–^^10^ though study findings are mixed.^11^^–^^13^

Recently, several epidemiological studies conducted in Caucasian populations provide a suggestive relationship between ambient air pollution and liver cancer incidence.^14^^,^^15^ Evidence based on the European Study of Cohorts for Air Pollution Effects (ESCAPE) project showed a positive association between various air pollutants (eg, PM_2.5_, NO_2_) and liver cancer incidence.^14^ VoPham et al prospectively examined the relationship between PM_2.5_ exposure and liver cancer risks by utilizing the Surveillance, Epidemiology, and End Results (SEER) database, which includes 16 United States cancer registries.^15^ They found exposure to baseline year PM_2.5_ was statistically associated with an increased risk of liver cancer in the follow-up period. A similar finding was also observed in a Taiwanese cohort study that additionally suggested chronic inflammation may mediate the effect of PM_2.5_ exposure in liver cancer risks.^16^

Existing evidence in different population showed exposure to metals could be attributed to the development of liver cancer. A study based on four European cohorts within the ESCAPE project found that PM_2.5_-bound metal constituents (eg, Fe and Ni) were associated with higher liver cancer risks, although the findings did not reach statistical significance.^14^ In a case-control study conducted in Europe, serum levels of copper (Cu), zinc (Zn), or Cu/Zn ratio showed a positive association with liver cancer.^17^ In Egypt, people who consumed food contaminated with heavy metals, including lead (Pb), cadminum (Cd), arsenic, or mercury (Hg), had a higher incidence of liver cancer compared with non-cancer patients.^18^

Given evidence suggesting an association of PM_2.5_ exposure with liver cancer^14^^–^^16^ and PM_2.5_ as a mixture of particles contributed by different sources, it is important to identify which specific components are associated with liver cancer because this relationship has not been investigated previously. Therefore, we opted to evaluate the association between exposure to metal constituents in PM_2.5_ and liver cancer risks in a Taiwanese cohort study.

## METHODS

### Study design and population

Recruitment of participants was primarily based on the REVEAL-HBV study (*n* = 23,820), a community-based cohort study in Taiwan (1991–1992), and participants were followed through December 31, 2014. The detailed study recruitment protocol was described in the previous study.^19^^–^^22^ In brief, we recruited a subpopulation of REVEAL-HBV based on participants whose residential address was located in the four counties (ie. New Taipei, Hsinchu, Chiayi, and Pingtung County) (*n* = 14,157) in the Taiwan Main Island. We primarily focused on these participants due to the rationale that the estimation of PM_2.5_ metal constituents is only applicable in these counties. Participants who had a non-geocoded address (*n* = 75) were excluded from the study. A further exclusion (*n* = 482) was made based on participants who had missing information as to smoking status, alcohol consumption, betel nut use, marital status, ethnicity, education, body mass index (BMI), serostatus of hepatitis B surface antigen (HBsAg), serostatus of anti-hepatitis C virus (HCV) antibody, serum alanine transaminase (ALT), or serum aspartate transaminase (AST). We further excluded four participants who had BMI >50.0 kg/m^2^ that may have resulted from an artificial error to ensure data reliability, as well as 82 female participants with a history of smoking or alcohol consumption. A total of 13,511 participants remained for the study analysis. We collected participants’ demographic information and lifestyle through structured questionnaires administered by public health nurses. A 10 mL peripheral blood sample was provided by each participant to test the serostatus of HBsAg and anti-HCV antibody. All participants agreed to be involved in the study with written informed consent, and the study protocols were approved by the Institutional Review Board (IRB) of National Yang-Ming University (IRB number: YM106108E).

### Ascertainment of hepatocellular carcinoma

At the study baseline period (1991–1992), participants with a history of HCC were excluded from the study based on health examination, history of HCC extracted from the questionnaire, or data linkage with the Taiwan Cancer Registry. Health examination, including abdominal ultrasonography and confirmatory diagnoses, was conducted among participants with a family history of HCC or cirrhosis who were seropositive for HBsAg or anti-HCV and had elevated serum levels of ALS, AST, or α-fetoprotein. Diagnosis of newly developed HCC in the study follow-up was ascertained using ultrasonography, serum α-protein, and computerized data linkage with the Taiwan Cancer Registry (ICD-O-FT = 1550 or 1551) and national health death certification system (ICD-9-CM = 1550 or 1551; ICD-10-CM = C220, C221, or C229) through December 31, 2014. The completeness of the Taiwan Cancer Registry was 92.8% in 2002, and this number was increased to 98.2% in 2014.

### Assessment for participants’ exposure to PM_2.5_ and PM_2.5_ metal constituents

A time-fixed exposure window was utilized for participants’ exposure to PM_2.5_ mass concentration and PM_2.5_ metal constituents. We estimated a 5-year average (2002–2006) concentration of air pollutants based on participants’ residential address provided in 1991–1992, and participants did not provide the information regarding changes of address in the follow-up period. A land-use regression (LUR) model was used to estimate participants’ exposure to PM_2.5_ in 2002–2006 as we described previously.^23^ The model’s temporal resolution was in a monthly basis with 250 m by 250 m grid. This approach coupled a LUR-based model with kriging techniques to predict spatial/temporal distribution of PM_2.5_. The performance of model prediction (ie, *R*-squared [*R*^2^]) was increased by 26–29% compared with the traditional LUR model, and the cross-validated *R*^2^ of hybrid kriging/land-use LUR model was 0.88 in the monthly model. In terms of constituents of PM_2.5_, a sampling network for annual concentration of PM_2.5_ components (ie, metals and soluble ions) was conducted in six geographical locations in Taiwan from 2002 to 2006. We extracted information on eight metals (Ba, Cu, Mn, Sb, Zn, Pb, Ni, and Cd) quantified using inductively coupled plasma mass spectrometry (ICP-MS). Details of the analytical methodology are available in our previous studies.^24^^,^^25^ The cross-validated *R*^2^ were 0.55 (Ba), 0.50 (Cu), 0.64 (Mn), 0.73 (Sb), 0.66 (Zn), 0.75 (Pb), 0.58 (Ni), and 0.45 (Cd). ArcGIS 10.2 software (Environmental Systems Research Institute, Redlands, CA, USA) with a Spatial Analyst module was used for participants’ address geo-coding, GIS layer extraction, and rendering prediction maps of PM_2.5_ metal constituents.

### Statistical analysis

To compare the differences in demographic information for participants with and without HCC events, we applied Fisher’s exact test for categorical variables and Wilcoxson Rank-sum test for continuous variables. A continuous fashion of PM_2.5_ metal constituents normalized by PM_2.5_ mass concentration was performed as the major exposure index throughout the analyses. In details, we first divided the metal constituents (ng/m^3^) by PM_2.5_ mass concentration (µg/m^3^), and this exposure index represented the concentration of metal constituents per 1 µg/m^3^ of PM_2.5_. The purpose of using this index was to tease out the effect of PM_2.5_ mass concentration on HCC incidence. In the next step, we transformed this exposure index using natural logarithm in order to make the distribution of index to be more symmetrical, which would minimize the influence of outlier values. We additionally applied a regular adjustment of PM_2.5_ mass concentration on the association of individual PM_2.5_ metal constituent with HCC incidence. Individual PM_2.5_ metal constituent and PM_2.5_ mass concentration were included in the same model.

We applied Cox proportional hazards models to evaluate the association between PM_2.5_ metal constituents and HCC incidence, and the follow-up year was selected as the time scale. Information of time-to-event for HCC participants was calculated from the enrollment date to the date of HCC diagnosis or liver cancer death, whichever came first. For censored participants, the follow-up year was calculated from the study entry date to the last day of study (for participants who neither develop HCC nor pass away) or the date of non-HCC death (for non-HCC death participants).

Hazard ratios (HRs) and 95% confidence intervals (CIs) for the association between long-term exposure to PM_2.5_ metal constituents and HCC risk were calculated with adjustments for age (30–39, 40–49, 50–59, or 60–65 years), sex (male or female), alcohol consumption habit (yes or no), smoking status (ever- or non-smoker), ALT levels (<15.0, 15.0 to 44.9, or ≥45.0 IU/L), serostatus of HBsAg (positive or negative), and serostatus of anti-HCV antibody (positive or negative) in the primary model (model 1). Additional adjustments for ethnicity (Hakka or Hoklo), marital status (married or not), BMI (continuous), and education (elementary school/illiterate, junior/senior high school, or college/university or higher) were made if applicable (model 2). To test the robustness of the study findings, we restricted the population among participants who were seronegative for HBsAg, anti-HCV, or both. Further analyses were performed by excluding HCC events that developed prior to 2006 to separate temporality of exposure to PM_2.5_ constituents (2002–2006) from that of HCC risk (2007–2014).

Since we opted to include eight correlated PM_2.5_ metal constituents (eFigure 1) in the Cox proportional hazard models to adjust for their mutual confounding, we applied a ridge regression approach to minimize the collinearity issue. Specifically, we introduced an *L2* penalty term for the regression coefficients of the metal constituents in the partial likelihood of the Cox proportional hazards model to stabilize their estimation. A similar *L2* regularization approach has been used to evaluate the impact of multiple environmental exposures on human health.^26^^–^^32^ The regression coefficients were estimated depending on the tuning parameter (λ) for penalization selected via five-fold cross-validation. The corresponding 95% CIs were calculated using 1,000 bootstrap resampling. All statistical analyses were performed by the R statistical program (version 3.5.1; R Foundation for Statistical Computing, Vienna, Austria). A two-side *P*-value of <0.05 was considered statistically significant.

## RESULTS

A total of 322 newly developed cases of HCC were ascertained with a median follow-up period of 23.1 years. Higher risk of HCC could be found among participants who were older, having higher BMI, seropositive for HBsAg, seropositive for anti-HCV antibody, or having a higher level of serum ALT at baseline period (Table 1). The median level of participants’ exposure to PM_2.5_ mass concentration was 32.5 µg/m^3^ (interquartile range, 9.4 µg/m^3^). PM_2.5_ containing metal constituents ranged from 0.64 to 465.2 ng/m^3^ among participants (Table 2). Participants who had HCC events were associated with higher exposure to PM_2.5_ Cu (*P* < 0.001), Mn (*P* < 0.001), Pb (*P* = 0.047), and Cd (*P* = 0.001) (Table 1).

**Table 1.** Baseline characteristics of participants in REVEAL-HBV at baseline period (1991–1992)

Variable	Censored (*n* = 13,189)		HCC events (*n* = 322)		*P*-value
	
	*n* (%)	mean ± SD	*n* (%)	mean ± SD	
**Age, years**					<0.001
30–39	3,640 (27.6)		37 (11.5)		
40–49	3,560 (27.0)		74 (23.0)		
50–59	4,384 (33.2)		145 (45.0)		
60–65	1,605 (12.2)		66 (20.5)		
**Sex, smoking, and drinking status**					<0.001
Female, non-drinker, and non-smoker	6,532 (49.5)		84 (26.1)		
Male, non-drinker, and non-smoker	2,575 (19.5)		82 (25.5)		
Male, non-drinker, and smoker	2,912 (22.1)		97 (30.1)		
Male, drinker, and non-smoker	245 (1.9)		12 (3.7)		
Male, drinker, and smoker	925 (7.0)		47 (14.6)		
**BMI, kg/m^2^**		23.9 ± 3.3		24.6 ± 3.6	<0.001
**Ethnicity**					0.002
Hoklo	7,020 (53.2)		199 (61.8)		
Hakka	6,169 (46.8)		123 (38.2)		
**Marital status**					0.71
Married	12,258 (92.9)		301 (93.5)		
Others	931 (7.1)		21 (6.5)		
**Education level**					0.37
Elementary School or Illiterate	8,374 (0.64)		230 (0.69)		
Junior or Senior High School	4,438 (0.34)		93 (0.28)		
College, University, or Higher	312 (0.02)		11 (0.03)		
**Seropositive for HBsAg**	2,102 (15.9)		173 (53.7)		<0.001
**Seropositive for anti-HCV antibody**	633 (4.8)		85 (26.4)		<0.001
**ALT, U/L**		12.22 ± 16.63		26.24 ± 33.47	<0.001
**Metals in PM_2.5_, ng/m^3^ (logarithm scale)**					
Ba		1.66 ± 0.53		1.69 ± 0.58	0.05
Cu		1.32 ± 1.27		1.53 ± 1.22	<0.001
Mn		1.56 ± 0.46		1.46 ± 0.53	<0.001
Sb		−0.41 ± 0.15		−0.41 ± 0.15	0.27
Zn		2.14 ± 1.21		2.01 ± 1.38	0.24
Pb		5.92 ± 0.16		5.93 ± 0.14	0.05
Ni		5.24 ± 0.60		5.21 ± 0.67	0.43
Cd		6.19 ± 0.51		6.28 ± 0.58	0.001
**PM_2.5_ mass concentration, µg/m^3^**		30.72 ± 5.24		30.66 ± 5.44	0.99

ALT, alanine aminotransferase; BMI, body mass index; HBsAg, hepatitis B-virus surface antigen; HCV, hepatitis C-virus; PM_2.5_, fine particulate matter.

**Table 2.** Exposure profiles of PM_2.5_ mass concentration and PM_2.5_-bound metals (2002–2006) among participants in REVEAL-HBV

	Median	Interquartile Range	Minimum	Maximum
**PM_2.5_, µg/m^3^**	32.5	9.4	13.2	44.4
**Metals in PM_2.5_, ng/m^3^**				
Ba	7.49	5.19	1.80	13.88
Cu	4.79	8.51	0.00	24.43
Mn	5.29	1.60	0.48	21.11
Sb	0.64	0.17	0.49	1.00
Zn	11.8	11.4	0.4	487.1
Pb	369.6	96.8	289.7	536.2
Ni	185.3	218.7	77.8	671.7
Cd	465.2	170.2	242.3	2,273.2

PM_2.5_, fine particulate matter.

We evaluated the association between single exposure to PM_2.5_ metal constituents and liver cancer risks. In model 1, with adjustment for the potential confounders, we found an elevated HCC risk was statistically associated with exposure to PM_2.5_ Cu (adjusted HR 1.13; 95% CI, 1.02–1.25) (model 1, Table 3). In addition, PM_2.5_ Ba and PM_2.5_ Zn were associated with liver cancer risk with marginal statistical significance (*P* < 0.1). PM_2.5_ Ba was positively associated with HCC risks (adjusted HR 1.30; 95% CI, 0.98–1.71), whereas PM_2.5_ Zn showed a negative relationship (adjusted HR 0.94; 95% CI, 0.87–1.01). Similar results were found for models further adjusted for marital status, BMI, and ethnicity (model 2, Table 3). By adding metal constituents and PM_2.5_ concentration in the same models, the PM_2.5_-HCC association were still robust for PM_2.5_ Ba and PM_2.5_ Cu (eTable 1). We also found a positive association between generic PM_2.5_ exposure (per 1 µg/m^3^ increment) and HCC incidence (HR 1.01; 95% CI, 0.99–1.04).

**Table 3.** Association between exposure to single PM_2.5_ metal constituent and incidence of liver cancer in REVEAL-HBV

Normalized Metals in PM_2.5_^c^	Model 1^a^		Model 2^b^	
	
	HR (95% CI)	*P*-value	HR (95% CI)	*P*-value
Ba	1.30 (0.98–1.71)	0.07	1.31 (0.97–1.77)	0.08
Cu	1.13 (1.02–1.25)	0.023	1.14 (1.02–1.29)	0.022
Mn	0.95 (0.75–1.22)	0.71	0.95 (0.71–1.27)	0.73
Sb	0.79 (0.48–1.30)	0.35	0.73 (0.44–1.21)	0.22
Zn	0.94 (0.87–1.01)	0.09	0.94 (0.87–1.02)	0.14
Pb	0.79 (0.30–2.09)	0.63	0.82 (0.31–2.17)	0.69
Ni	0.90 (0.77–1.04)	0.15	0.91 (0.78–1.06)	0.22
Cd	0.93 (0.79–1.09)	0.37	0.92 (0.78–1.07)	0.30

CI, confidence interval; HR, hazard ratio; PM_2.5_, fine particulate matter.

^a^Model 1 was adjusted for age, sex, smoking status, alcohol consumption, serum alanine transaminase, seropositive for hepatitis B virus surface antigen, and seropositive for anti-hepatitis C virus antibody.

^b^Model 2 was model 1 with additional adjustment for marital status, body mass index, education, and ethnicity.

^c^Metals (ng/m^3^) were normalized by PM_2.5_ mass concentration (µg/m^3^) followed by natural logarithm-transformation.

**Figure 1.**
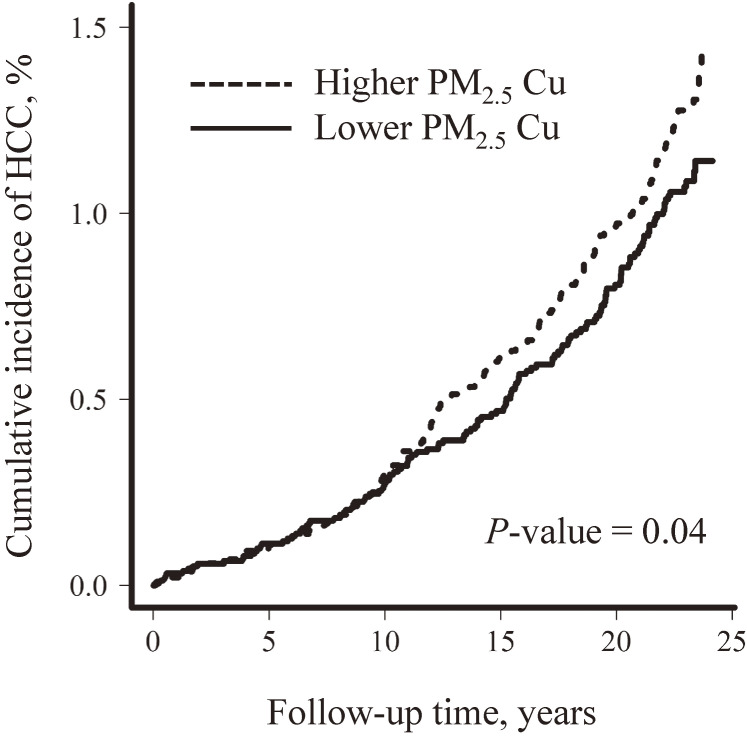
Cumulative incidence of liver cancer by exposure to cu in fine particulate matters. PM_2.5_ Cu exposure level was dichotomized by the normalized median value (−8.61, natural-logarithm scale). The *P*-value in Nelson-Aalen curve was 0.04 using the log rank test. The model was adjusted for age, sex, alcohol consumption, smoking status, serostatus of HBV, serostatus of anti-HVC antibody, and serum alanine transaminase. anti-HCV, anti-hepatitis C virus antibody; HBV, hepatitis B virus; PM_2.5_, fine particulate matter.

Figure 1 displayed the time trend of cumulative incidence of HCC stratified by participants’ long-term exposure to PM_2.5_ Cu levels. A higher PM_2.5_ Cu exposure (above or equal to the normalized median value [−8.61]) was statistically associated with an elevated HCC incidence compared with the reference group (below normalized median value) (*P* = 0.04). The HCC cumulative curves for both groups (higher or lower exposure) were diverged after 10 to 12 years of follow-up.

We utilized a ridge regression approach to evaluate this relationship. After minimizing the mutual confounding arising from co-exposure to metal components in PM_2.5_, we found PM_2.5_ Cu was consistently associated with an increased risk of liver cancer. The adjusted hazard ratio was 1.06 (95% CI, 1.00–1.13) with statistical significance. The marginal significant relationships for PM_2.5_ Ba-HCC and Zn-HCC in the single exposure setting (model 1, Table 4) were diminished to the null association after controlling for co-exposure to other PM_2.5_ metal constituents. This finding could be partially explained by the high correlation between PM_2.5_ Ba and PM_2.5_ Zn (Spearman’s correlation coefficient = −0.80, eFigure 1).

**Table 4.** Multiple exposure to metals in PM_2.5_ and incidence of liver cancer

Normalized metals in PM_2.5_^a^	Hazard ratio (95% confidence intervals)^b^
Ba	1.01 (0.99–1.03)
Cu	1.06 (1.00–1.13)
Mn	0.99 (0.96–1.03)
Sb	0.99 (0.97–1.01)
Zn	0.97 (0.92–1.01)
Pb	1.00 (0.99–1.01)
Ni	0.99 (0.96–1.02)
Cd	0.99 (0.95–1.05)

PM_2.5_, fine particulate matter.

^a^Metals (ng/m^3^) were normalized by PM_2.5_ mass concentration (µg/m^3^) followed by natural logarithm transformation.

^b^A Cox proportional hazards model in ridge regression setting (*L2* penalty) was utilized. Models were adjusted for age, sex, smoking status, alcohol consumption, serum alanine transaminase, seropositive for hepatitis B virus surface antigen, seropositive for anti-hepatitis C virus antibody, and multiple metal exposures.

In Table 5, we performed sensitivity analysis by restriction on participants who were seronegative to hepatitis virus infection (ie, HBV, HCV), developed liver cancer after 2006, or both. The positive association between PM_2.5_ Cu and HCC remained, although statistical insignificance was found in some sub-populations.

**Table 5.** Sub-group analysis for association between exposure to PM_2.5_ Cu and incidence of liver cancer

HCC event period	Restriction on HBV and HCV serostatus	HCC events	Sample size	HR (95% CI)^a^	*P*-value
1991–2014	No restriction	322	13,511	1.13 (1.02–1.25)	0.023
1991–2014	Seronegative for HBV	149	11,236	1.12 (0.95–1.30)	0.17
1991–2014	Seronegative for anti-HCV antibody	237	12,793	1.18 (1.04–1.34)	0.009
1991–2014	Seronegative for HBV and anti-HCV antibody	76	10,620	1.15 (0.92–1.43)	0.23
2007–2014	No restriction	156	12,102	1.10 (0.95–1.28)	0.18
2007–2014	Seronegative for HBV	84	10,122	1.13 (0.91–1.39)	0.28
2007–2014	Seronegative for anti-HCV antibody	108	11,528	1.08 (0.91–1.28)	0.40
2007–2014	Seronegative for HBV and anti-HCV antibody	41	9,593	1.04 (0.78–1.68)	0.79

anti-HCV, anti-hepatitis C virus antibody; CI, confidence interval; HBV, hepatitis B virus; HCC, hepatocellular carcinoma; HR, hazard ratio; PM_2.5_, fine particulate matter.

^a^Models were adjusted for age, sex, smoking status, alcohol consumption, serum alanine transaminase, seropositive for HBV surface antigen (if applicable), and seropositive for anti-HCV antibody (if applicable).

## DISCUSSION

This study found long-term exposure to Cu constituent in PM_2.5_ was associated with an increased risk of liver cancer. The findings remained statistically significant after controlling for co-exposure to other PM_2.5_ metal components. Sensitivity analysis restricting the non-hepatitis population, excluding liver cancer development prior to the exposure assessment period (ie, 2002–2006), or both did not substantially change the positive association.

Large cohort studies conducted in Europe, the United States, and Taiwan provide evidence linking exposure to PM_2.5_ and risk of liver cancer.^14^^–^^16^ Pedersen et al found a 5 µg/m^3^ increment on PM_2.5_ exposure was associated with a 34% increased risk of liver cancer in the meta-analysis, and findings were consistent across different air pollutants (eg, NO_2_, PM_10_).^14^ Cu constituent in PM_10_ was also positively associated with liver cancer risk, with a HR of 1.42 (95% CI, 0.92–2.21) per 20 ng/m^3^ increment.^14^ Although the Cu component in PM_2.5_ did not show a consistent association with HCC incidence, it could be partially due to the better model prediction capability of PM_10_ Cu (average of LOOCV *R*^2^ = 0.73) compared to PM_2.5_ Cu (average of LOOCV *R*^2^ = 0.65). The non-differential exposure misclassification of PM_2.5_ Cu may attenuate its association with HCC incidence. A study based on the United States cancer registries revealed a significant relationship between PM_2.5_ exposure and HCC incidence. The HCC incidence was increased by 26% with 10 µg/m^3^ increment on PM_2.5_. A recent study in six European cohorts found that NO_2_ and black carbon (BC) robustly associated with liver cancer incidence in two-pollutant models.^33^ Most metal constituents (ie, Cu, Fe, Zn, S, Ni, and V) of PM_2.5_ were positively association with liver cancer incidence with adjustment for PM_2.5_. The positive Cu-HCC association was consistent with our major findings.

In addition, some evidence was found for the association of PM_2.5_ containing metals with the risk of lung cancer.^30^ Findings based on a consortium including 14 European cohort studies showed exposure to Cu constituent in PM_2.5_ was positively associated with the risk of lung cancer among participants whose residential address had not changed (HR 1.25; 95% CI, 1.01–1.53 per 5 ng/m^3^). Additionally, the ambient PM_2.5_ Cu has been referred to anthropogenic sources, such as brake wear^31^^,^^32^ or copper smelting.^33^^,^^34^ Chen et al found Cu smelting industries and vehicular emission were major sources (13.1%) for fine-sized metals in central Taiwan.^35^ Another study conducted in Taiwan found that PM_2.5_-bound Cu and other industrial-related metals (eg, Fe, Zn) was higher in the industrial site in a typical heavy-industrial city using positive matrix factorization (PMF).^36^ This evidence suggested that the PM_2.5_-bound Cu was primarily generated by industrial or traffic-related sources. Taken together, the evidence suggests a potential link between PM_2.5_ Cu and liver cancer, where the traffic exposure or smelting industry could be the major contributors.

Evidence based on human subjects suggests inflammation may serve as the underlying mechanism linking PM_2.5_ Cu exposure and liver cancer development. Observational studies found exposure to particulate matter was associated with an increase in liver inflammation marker (eg, ALT),^37^^–^^39^ which has been shown to be the risk factor of liver cancer.^40^ Our previously finding also suggested chronic inflammation may mediate the effect of PM_2.5_ exposure on liver cancer development.^16^ A more recent study based on healthy participants in a crossover design demonstrated a 5-hour exposure to brazing fumes containing copper and zinc significantly induced serum levels of c-reactive protein (CRP), a global marker for inflammation.^41^ In addition, experimental animal studies provide supporting evidence for PM_2.5_ Cu-inflammation relationship. In rats, exposure to copper oxide nanoparticles upregulated gene expression related to pro-inflammatory markers and cell proliferation/survival in lung tissues.^42^ More evidence in chicken and zebrafish revealed a possible interplay between inflammation and oxidative stress induced by copper exposure.^43^^–^^45^

To correctly interpret the study findings, several caveats regarding study limitations should be considered. First, we assumed PM_2.5_ metal constituents assessed in 2002–2006 could serve as a surrogate for participants’ long-term exposure profile. Since the participants were recruited in the early 1990’s with a more than 20-year follow-up, it is likely the exposure profile of PM_2.5_ components may change over time. Due to the lack of historical measurements of metal constituents in PM_2.5_ prior to 2002, we were unable to verify the concordance between the LUR-estimated (2002–2006) and historical (1991–2001) levels of PM_2.5_ constituents. Also, the lack of participants’ residential address in the follow-up period may introduce certain levels of exposure misclassification. Second, participants’ exposure to PM_2.5_ metal constituents was primarily based on a LUR model that usually has better prediction for the spatial distribution of air pollutants compared with a temporal prediction capacity. However, sensitivity analysis based on participants who developed HCC after 2007 consistently showed a positive PM_2.5_ Cu-HCC association, and it provided evidence of study robustness. Third, participants’ exposures to PM_2.5_ constituents were based on their residential address linking to ambient pollution levels estimated LUR techniques, and their personal exposure levels were lacking. Also, a *R*-squared of 0.50 on PM_2.5_ Cu estimation may raise the concern of exposure uncertainty. Therefore, we may have introduced exposure misclassification from the personal activity pattern (eg, hours staying at home, workplace, or transportation). This may bias our finding toward the null association given the nature of non-differential misclassification. Fourth, we did not collect metal exposure through other routes, such as occupational exposure or dietary sources, so the possibility of un-measured confounding could not be excluded. However, ethnicity background (Hakka or Hoklo) that partially reflects dietary pattern was adjusted in the analysis, and it may reduce confounding due to metal exposure from dietary routes.

Fourth, information of some known risk of HCC (eg, Wilson diseases, diabetes, parasites) was not collected in this study, which may introduce the issue of un-measured confounding. Either a positive or negative confounding bias would be possible depending on the relationship between these factors and PM_2.5_-bound metal constituents. Therefore, over- or under-estimation on PM_2.5_ Cu-HCC association are both possible.

Last, it is known long-term exposure to PM_2.5_ can contribute to cardiovascular diseases,^46^^–^^48^ and consequently we cannot rule out the likelihood of competing risk due to cardiovascular death. On the contrary, several strengths of this study should be mentioned, including a long follow-up period, spatial interpolation on exposure assessment, and a large sample size.

### Conclusion

In conclusion, we found long-term exposure to PM_2.5_ containing Cu was positively associated with liver cancer risks. The findings remained consistent with the adjustment for co-exposure to other PM_2.5_ metal components. Additional epidemiological studies are needed to confirm this finding, and toxicological experiments should be conducted to elucidate the PM_2.5_ Cu-HCC etiology.

## SUPPLEMENTARY MATERIAL

eTable 1. Association between exposure to single PM_2.5_ metal constituent and incidence of liver cancer in REVEAL-HBV with regular PM_2.5_ adjustment

eFigure 1. Pairwise Spearman’s correlation coefficients of metals in fine particulate matter (2002–2006) in REVEAL-HBV
