# Supplementary material for: Exposure to PM2.5 Metal Constituents and Liver Cancer Risk in REVEAL-HBV
Source: J Epidemiol. 2024 Feb 5;34(2):87–93. doi: 10.2188/jea.JE20220262 (PMC10751193; doi:10.2188/jea.JE20220262)

eTable 1. Association between exposure to single $PM_{2.5}$ metal constituent and incidence of liver cancer in REVEAL-HBV with regular $PM_{2.5}$ adjustment

	**Model 1[a]**		**Model 2[b]**	
**Metals in $PM_{2.5}$[c]**	**HR (95% CI)**	***P*-value**	**HR (95% CI)**	***P*-value**
Ba	1.40 (0.84–2.33)	0.20	1.36 (0.77–2.38)	0.29
Cu	1.11 (1.00–1.25)	0.05	1.13 (1.00–1.28)	0.05
Mn	0.94 (0.73–1.20)	0.60	0.90 (0.67–1.20)	0.48
Sb	1.03 (0.50–2.11)	0.94	0.88 (0.41–1.90)	0.75
Zn	0.95 (0.86–1.04)	0.24	0.96 (0.87–1.05)	0.36
Pb	1.15 (0.30–4.40)	0.84	1.32 (0.79–1.12)	0.69
Ni	0.92 (0.77–1.09)	0.34	0.94 (0.79–1.12)	0.49
Cd	1.08 (0.78–1.49)	0.64	1.15 (0.72–1.85)	0.56

CI, confidence interval; HR, hazard ratio.

[a] Model 1 was adjusted for age, sex, smoking status, alcohol consumption, serum alanine transaminase, seropositive for HBV surface antigen, seropositive for anti-HCV antibody, and $PM_{2.5}$ mass concentration.

[b] Model 2 was Model 1 with additional adjustment for marital status, BMI, education, and ethnicity.

[c] Metals (ng/m^3) were natural-logarithm transformed.

eFigure 1. Pairwise Spearman's correlation coefficients of metals in fine particulate matter (2002–2006) in REVEAL-HBV

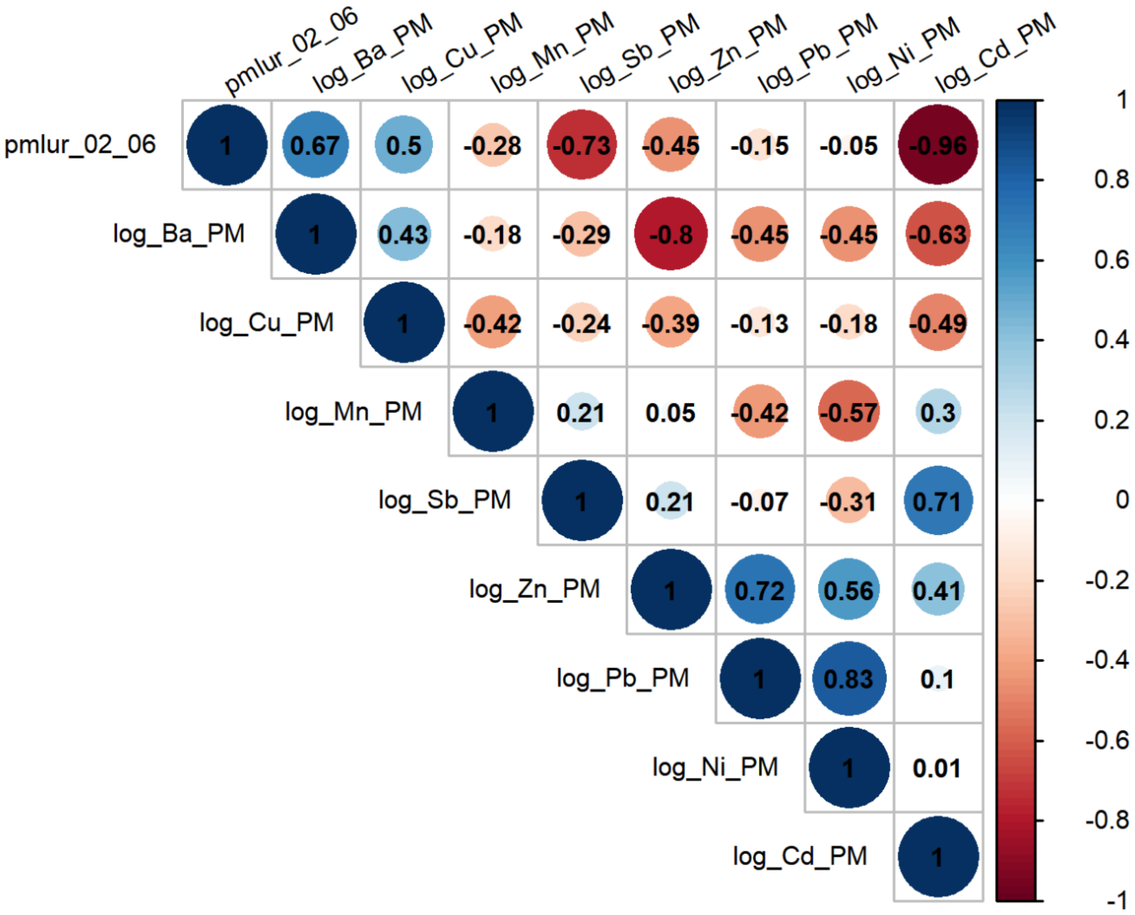

Supplement: Supplementary file 1 [file je-34-087-s001.pdf]
